# Impact of a School-Based Gardening, Cooking, Nutrition Intervention on Diet Intake and Quality: The TX Sprouts Randomized Controlled Trial

**DOI:** 10.3390/nu13093081

**Published:** 2021-09-01

**Authors:** Matthew J. Landry, Alexandra E. van den Berg, Deanna M. Hoelscher, Fiona M. Asigbee, Sarvenaz Vandyousefi, Reem Ghaddar, Matthew R. Jeans, Lyndsey Waugh, Katie Nikah, Shreela V. Sharma, Jaimie N. Davis

**Affiliations:** 1 Stanford Prevention Research Center, School of Medicine, Stanford University, Palo Alto, CA 94305, USA; matthewlandry@stanford.edu; 2 Michael & Susan Dell Center for Healthy Living, Department of Health Promotion and Behavioral Sciences, The University of Texas Health Science Center at Houston (UTHealth), Austin Campus, Austin, TX 78701, USA; alexandra.e.vandenberg@uth.tmc.edu (A.E.v.d.B.); deanna.m.hoelscher@uth.tmc.edu (D.M.H.); 3 Department of Nutritional Sciences, College of Natural Sciences, The University of Texas at Austin, Austin, TX 78723, USA; fiona.asigbee@austin.utexas.edu (F.M.A.); reemghaddar94@gmail.com (R.G.); mjeans@utexas.edu (M.R.J.); katienikah@utexas.edu (K.N.); 4 Department of Pediatrics, Bellevue Hospital, Grossman Medical Center, New York University, New York, NY 10016, USA; sarvenaz.vandyousefi@nyulangone.org; 5 Sprouts Healthy Communities Foundation, Phoenix, AZ 85054, USA; lyndseywaugh@sprouts.com; 6 Department of Epidemiology, Human Genetics, and Environmental Sciences, The University of Texas Health Science Center at Houston (UTHealth), Houston, TX 77030, USA; shreela.v.sharma@uth.tmc.edu

**Keywords:** diet quality, school-based intervention, gardening, cooking, nutrition, low-income, children, nutrition education

## Abstract

School gardens have become common school-based health promotion strategies to enhance dietary behaviors in the United States. The goal of this study was to examine the effects of TX Sprouts, a one-year school-based gardening, cooking, and nutrition cluster randomized controlled trial, on students’ dietary intake and quality. Eight schools were randomly assigned to the TX Sprouts intervention and eight schools to control (i.e., delayed intervention) over three years (2016–2019). The intervention arm received: formation and training of Garden Leadership Committees; a 0.25-acre outdoor teaching garden; 18 student lessons including gardening, nutrition, and cooking activities, taught weekly in the teaching garden during school hours; and nine parent lessons, taught monthly. Dietary intake data via two 24 h dietary recalls (24 hDR) were collected on a random subsample (*n* = 468). Dietary quality was calculated using the Healthy Eating Index 2015 (HEI-2015). The intervention group compared to control resulted in a modest increase in protein intake as a percentage of total energy (0.4% vs. −0.3%, *p* = 0.021) and in HEI-2015 total vegetables component scores (+4% vs. −2%, *p* = 0.003). When stratified by ethnicity/race, non-Hispanic children had a significant increase in HEI-2015 total vegetable scores in the intervention group compared to the control group (+4% vs. −8%, *p* = 0.026). Both the intervention and control groups increased added sugar intake; however, to a lesser extent within the intervention group (0.3 vs. 2.6 g/day, *p* = 0.050). School-based gardening, cooking, and nutrition interventions can result in significant improvements in dietary intake. Further research on ways to scale and sustain nutrition education programs in schools is warranted. The trial is registered at ClinicalTrials.gov (NCT02668744).

## 1. Introduction

According to the 2020–2025 Dietary Guidelines for Americans, persons should consume fruits and vegetables as part of a healthy eating pattern to reduce their risk for diet-related chronic diseases, such as cardiovascular disease, type 2 diabetes, some cancers, and obesity [1]. Childhood is a critical period during which lifelong eating habits are established, influencing future risk for diet-related chronic diseases [2,3]. Despite these recommendations, the vast majority of children in the US continue to consume energy-dense, nutrient-poor diets [3,4,5,6,7]. A multi-year cross-sectional investigation of dietary recalls from children and adolescents aged 2–19 years from the National Health and Nutrition Examination Survey (NHANES) cycles (1999–2016) found that dietary quality as measured by the Healthy Eating Index-2015 (HEI-2015) increased 11.2%; however, within children aged 6–11 years, 52.5% still had poor dietary quality in 2015–2016 [3].

Elementary-aged children consume between one-third and one-half of their daily calories at school, making schools a crucial setting for interventions that alter their food environment and promote healthful behaviors [8]. Currently, 78% of states require nutrition education for all students; however, few states provide training or curricula to do this [9]. School garden interventions have become popular among schools as a modifiable aspect of the school environment that can be used to educate students on nutrition and healthy eating habits. A longitudinal study of US elementary schools between 2006 and 2014 found that the prevalence of gardens increased from 11.9% in 2006–2007 to 31.2% in 2013–2014 [10]. As school garden advocates push for more gardens in schools, they often cite the wealth of direct and indirect benefits that school gardens can have on students, including academic performance, psychosocial factors, dietary intake, mental health, obesity risk factors, and physical activity [11,12,13,14,15,16,17,18,19,20]. One of the most commonly reported benefits is an increase in fruit and vegetable intake. In 2017, a review of gardening interventions found that 10 of the 14 included studies showed significant increases in fruit and vegetable intake [21]. Similarly, an umbrella review of garden-based interventions in children ages 6 years and younger found that gardening interventions were effective at improving nutrition-related outcomes, particularly fruit and vegetable consumption [22]. A primary limitation of most previous studies is that they did not conduct randomization of either children or schools, increasing the risk for selection bias and systematic differences between study groups [21]. This limits the ability to determine whether causality exists between changes in dietary behaviors and garden-based interventions. The few existing randomized controlled trials (RCTs) have been limited by short duration, intervention intensity and fidelity of implementation, and methods used to assess overall dietary or vegetable intake (e.g., food frequency questionnaires) [14,18,23,24].

Therefore, the overall goal of TX Sprouts, a gardening, cooking, and nutrition program, cluster RCT in elementary schools, was to examine the effects this program on 3rd–5th grade children’s dietary intake, obesity outcomes, and metabolic health biomarkers. Results of the TX Sprouts impact on dietary intake (from a vegetable screener), obesity, and blood pressure have been previously published [25]. This RCT produced additional data on dietary psychosocial variables via survey, academic performance, physical activity via accelerometers, diabetes and metabolic biomarkers via fasting blood draws, and skin carotenoids via resonance Raman spectroscopy; results of these outcomes are forthcoming. Specifically, this paper focuses on improvements in dietary intake and quality using 24 h dietary recalls (24 hDR) data collected on a random subsample of children. We also sought to examine if there was an interaction between intervention group and child ethnicity/race. We hypothesized that children in the TX Sprouts intervention compared to the control group would have improved dietary intake and quality. We also hypothesized that there would be an interaction between intention-to-treat effects and child ethnicity/race, with Hispanic children having greater improvements in dietary intake and quality compared to non-Hispanic children.

## 2. Materials and Methods

### 2.1. Study Design and Participants

The complete design and methodology of the TX Sprouts study have been previously published [26]. Briefly, this is a school-based cluster randomized controlled trial where 16 elementary schools were randomly assigned to either: (1) TX Sprouts Intervention (n = 8 schools) or (2) Control (delayed intervention; *n* = 8 schools). The intervention was implemented in three schools per arm in the 2016–2017 (*n* = 6 total) and 2017–2018 (*n* = 6 total) school years, and two schools per arm in the 2018–2019 academic year (*n* = 4 total). All schools had to meet the following inclusion criteria: (1) high proportion enrollment of Hispanic children (>50%); (2) high proportion of children participating in the free and reduced-price lunch (FRL) program (>50%); (3) location within 60 miles of the University of Texas at Austin (UT-Austin) campus; and (4) no existing garden or gardening program. The trial was registered at ClinicalTrials.gov (NCT02668744).

All 3rd–5th grade students and parents at the recruited schools were contacted to participate in TX Sprouts via information tables at “Back to School” and “Meet the Teacher” evening events, flyers sent home with students, and class announcements from teachers. Students and their parents signed written assent and consent forms, respectively. While all 3rd–5th grade students at participating schools attended the TX Sprouts classes in the garden as part of their in-school curriculum, only students with signed assent/consent forms participated in the measurements. All recruitment materials were available in both English and Spanish and were approved by the Institutional Review Board at the University of Texas at Austin (IRB#2014-11-0045), as well as the research departments at each of the participating school districts.

### 2.2. Description of TX Sprouts Intervention

The complete description of the TX Sprouts intervention has been described at length elsewhere [25,26]. Briefly, the TX Sprouts program was based on the social ecological model, which treats the child as nested within immediate contexts or micro-systems (e.g., school, family, community) that reciprocally interact with each other and the child over time to shape development and behaviors [27].

A 0.25-acre outdoor teaching garden was built at every intervention school in the spring prior to the academic year of baseline measurements. All gardens included: raised vegetable beds; in-ground native and herb beds; a large shed for tools and materials; a whiteboard; and seating for classes. The schools were provided with the materials and supplies needed for garden upkeep (e.g., rakes, hoses, etc.) and for teaching the lessons, (e.g., tables, chairs/benches, cooking grill, portable hand-washing sink, pots/pans, etc.). Vegetables, fruit, and herbs planted in the garden were chosen based upon seasonality, soil type, and usage of recipes used in the curriculum, but often included culturally specific produce such as squash, peppers, and cilantro.

Garden Leadership Committees (GLC) were formed at each intervention school and were comprised of interested stakeholders, such as teachers, parents, community members, school staff, and students. GLCs assisted with the following: (a) physical garden design and build; (b) hosting several garden workdays; and (c) development and implementation of long-term garden maintenance and sustainability plan.

Full-time experienced and trained nutrition and garden educators taught 18 one-hour TX Sprouts lessons separately to each 3rd–5th grade class throughout the school year as part of their normal school day. The TX Sprouts curriculum was adapted from LA Sprouts [28] and Junior Master Gardener, a program developed by the Texas A&M AgriLife Extension Service [29]. TX Sprouts lessons were designed to improve a variety of diet-related psychosocial behaviors, including nutrition, gardening, and cooking knowledge, self-efficacy and attitudes, and increase a child’s willingness to try and preference for fruits and vegetables, all of which would lead to increased fruit and vegetable intake and decreased intake of added sugar and refined grains. Every lesson included either a garden taste-test (seven lessons) or a cooking activity (11 lessons) and a sampling of different “aguas frescas,” which are flavored/infused waters with no added sugar. The student curriculum was designed to be culturally tailored to Hispanic children, including culturally appropriate recipes, content, and activities [30]. The control schools received a delayed intervention in the year after the post-testing for that wave.

Optional sixty-minute lessons were taught monthly, for a total of nine lessons throughout the school year. The parents’ curriculum was adapted from the LA Sprouts program [28] and paralleled the nutrition and gardening topics/activities taught to the children [26]. The parent curriculum included the following topics: importance of family eating, healthy shopping, and increasing home available and access of healthy foods. The parent curriculum was taught in both English and Spanish by the same garden/nutrition educators teaching the students. The dates and times of classes varied widely across school sites, and parent classes were offered in mornings, during school hours, after-school hours, evenings, and on weekends to account for parent preferences and schedules at the various school sites. Parents were incentivized to attend the lessons with free meals, produce giveaways, groceries, water bottles, t-shirts, garden gloves, raffles for gift cards, and free childcare for children and siblings. The lessons were advertised and promoted by posting flyers, sending home newsletters, and sending out reminder text messages. Dose, reach, and fidelity of the intervention (student and parent classes) are reported elsewhere [25].

### 2.3. Outcome Measurements

Data were collected on children and parents at baseline (within the first month of the academic school year) and at follow-up (within the last month of the academic school year) at intervention and control schools. A full description of all measurements collected is published elsewhere [26]. Children were asked questions about their age, grade, and sex on a survey. Parents were asked to complete a questionnaire packet, which was either provided at recruitment events or sent home to them with their child.

### 2.4. Collection of Dietary Data via 24-h Dietary Recalls

Primary dietary intake was assessed using two unannounced 24 hDR within a two-week window of baseline and follow-up data collection time points. At baseline, sixteen students (eight male and eight female) were randomly selected from each grade level at each school to be contacted for recalls, for a total of 48 students per school. If any of the students were not available or did not want to participate in recalls, then additional students were randomly selected as back-ups. Efforts were made to collect one weekday and one weekend recall; however, in some cases students completed two weekday recalls or two weekend recalls.

Data were collected using Nutrition Data Systems for Research (NDS-R, 2016 version), a computer-based software application developed at the University of Minnesota Nutrition Coordinating Center (NCC). Dietary recalls were collected in a standardized fashion using a multiple-pass interview approach consisting of five steps to ensure completeness and accuracy [31,32]. Five distinct passes provided multiple opportunities for the student to recall food intake. Students took approximately 20 to 30 min to complete each recall. A Food Amounts Booklet was distributed to students at school before recalls began and was used to help estimate serving sizes during recalls. To further aid in collecting recalls, school breakfast and lunch menus and portion sizes were obtained from school food services. Parents and/or guardians of students were allowed to assist with recalling food items consumed and estimated serving sizes as needed. Each student received a $10 gift card or $10 check for completing both recalls at baseline and both recalls at follow-up, totaling up to $20 in incentives.

All data collectors were trained by NDS-R certified lead staff and were blinded to a student’s intervention group. The lead dietary assessment nutritionist conducted a quality check after each of the study’s data collection time points, which involved an in-depth review of both individual and composite NDS-R reports for completeness and errors.

### 2.5. Calculation of the Healthy Eating Index-2015 (HEI-2015)

Diet quality was assessed using the Healthy Eating Index-2015 (HEI-2015). The HEI-2015 is a valid and reliable composite measure that assesses overall diet quality and compliance with the DGA-2015 [33,34]. The index is appropriate for examining diet quality of the U.S. population as well as specific subgroups, such as children and adolescents or racial-ethnic populations, and has a wide range of applications including epidemiology, population monitoring and surveillance, and nutrition interventions [35]. The HEI-2015 includes 13 components (total fruits, whole fruits, total vegetables, greens and beans, whole grains, dairy, total protein foods, seafood and plant proteins, fatty acids, refined grains, sodium, saturated fats, and added sugars) [36]. The first nine components are adequacy scores, with higher scores indicating higher consumption, and scores of 0 indicating no intake. The remaining four components (refined grains, sodium, saturated fat, and added sugars) are moderation components for moderation. For these components, reverse scoring is applied, with higher scores indicating lower consumption. Total fruits, whole fruits, total vegetables, greens and beans, total protein foods, seafood and plant proteins have a maximum score of 5, and whole grains, dairy, fatty acids, refined grains, sodium, saturated fats, and added sugars have a maximum score of 10. A total HEI score is derived by adding up the 13 component scores. The maximum total HEI score is 100 and signifies the highest possible compliance to the DGA. HEI scores were calculated using an average of each participant’s dietary recalls. Because two dietary recalls were used for each participant, scores were calculated by summing across both days per participant before applying the HEI scoring standards and performing further analyses. HEI scores were calculated using a freely available SAS code developed by the University of Minnesota Nutrition Coordinating Center [37].

### 2.6. Selection of Nutrients for Analysis

In addition to total energy intake and gram and percent of total energy intake from each of the three macronutrients (carbohydrates, fats, and protein), selected nutrients were chosen for analysis based on designation as a “shortfall nutrient,” nutrient of public health concern, or nutrient to reduce or limit consumption of based on recommendations of the 2015–2020 Dietary Advisory Committee (DGAC) [38]. The 2015–2020 DGAC determined that several nutrients: vitamins A, E, and C; folate; magnesium; and iron (in adolescent females), were under-consumed relative to the Estimated Average Requirement or Adequate Intake and were designated as “shortfall nutrients.” Fiber, calcium, vitamin D, and potassium were confirmed as nutrients of public health concern due to their underconsumption being linked to adverse health outcomes. Nutrients to reduce or limit consumption of were saturated fatty acids, added sugars, and sodium.

### 2.7. Statistical Analysis

Study data were collected and managed using REDCap (Research Electronic Data Capture) hosted at UT-Austin [39,40]. Basic summary statistics (frequencies, percentages, means, standard deviations) were used to describe the sociodemographic characteristics of students in intervention and control groups. Chi-square (X^2^) tests and univariate analyses of variance (ANOVA) were used to determine differences in participant characteristics between control and intervention groups. Prior to multivariable analysis, intraclass correlation coefficients (ICC) were obtained for each outcome of interest to determine the extent of school-level clustering. The ICCs were low (<0.02) and within range of previously reported values of school-level ICC [41]. Random-effects models were employed for all outcomes to account for potential clustering. Repeated measures analyses of covariance (ANCOVA) were run to examine changes in macronutrients, shortfall micronutrients, micronutrients of public health concern, nutrients to reduce consumption, and HEI-2015 total and component scores, between control and intervention groups. Interactions between intervention group and child ethnicity/race were tested. In addition to school-level clustering, models were adjusted for baseline dietary intake, sex, child ethnicity/race, change in energy intake (kcal). We are reporting the results of the trial using complete case analysis. Children who provided complete data for demographics, anthropometrics, and 24 h diet recalls were used for the complete case analysis, and therefore a fixed sample size was used under this assumption. Data were analyzed in RStudio (Version 1.2.5042, RStudio Team, 2020, PBC, Boston, MA, USA).

## 3. Results

Of the 4239 eligible children at the 16 schools, 3302 children (78%) consented to be in the study. Of those consented, 3135 children (74% of eligible children or 95% of those consented) completed baseline clinical measurements and child surveys and were included in the clinical trial. The intervention group included 1412 children (or 45%) and the control group included 1723 children. Of the 1121 students randomly selected for 24 hDRs, (69%) (*n* = 738 children; *n* = 361 intervention group, *n* = 377 control group) completed both recalls at baseline. At follow-up, 63% (*n* = 468 children; *n* = 234 intervention group, *n* = 234 control group) completed two 24 hDRs.

The average age of children at baseline was 9.8 years and 48% were male. Approximately 55% were Hispanic, and the average percentage of children receiving free or reduced-price breakfast/lunch at school was 59%. There were no significant differences in any of the sociodemographic variables (age, gender, ethnicity/race, and participation in the free and reduced lunch program or family participation in the Supplemental Nutrition Assistance Program) between children who completed 24 hDRs and those who did not. Additionally, there were no significant differences in any sociodemographic variables between children in intervention versus control groups within the sub-sample of children who completed 24 hDRs (data not shown).

At baseline, average energy intake was 1464 ± 558 kcals with carbohydrates, protein, and fat providing 50%, 17%, and 33% of total energy, respectively. Pre- and post-intervention and absolute change means and standard deviations as well as intention-to-treat effects of the intervention on macronutrients, shortfall micronutrients, micronutrients of public health concern, and nutrients to reduce consumption are shown in Table 1. Compared to students in the control condition, children in the intervention showed a modestly increased protein intake as a percent of total energy (0.4% vs. −0.3%, *p* = 0.021). There was no statistically significant impact of the intervention on any shortfall nutrients or micronutrients of public health concern. Both the intervention and control groups increased added sugar intake; however, the intervention group did increase to a lesser extent compared to the control (0.3 vs. 2.6 g/day, *p* = 0.050). There were no significant differences between groups when examining the interaction effect of ethnicity/race on intention-to-treat.

At baseline, average dietary quality was comparable to the US average, with an average HEI total score of 52.9 ± 12.4 (out of a possible score of 100). Pre- and post-intervention and absolute change means and standard deviations as well as intention-to-treat effects of the intervention on HEI-2015 component scores are shown in Table 2. The intervention group had increased HEI-2015 total vegetables component scores (+0.2 vs. −0.1, *p* = 0.003) compared to the control group. There was also a significant interaction effect between intervention group and ethnicity/race for total vegetables (*p* = 0.033). When stratified by ethnicity/race, Hispanic children in the intervention group compared to the control group did not have significantly different HEI-2015 total vegetable component scores (−0.1 vs. +0.1, *p* = 0.886). Among non-Hispanic children, there was a statistically significant increase in total vegetable scores in the intervention group compared to control group (+0.2 vs. −0.4, *p* = 0.026). Changes in intervention compared to control groups were not significantly significant for any other HEI-2015 component score or for HEI-2015 total score.

## 4. Discussion

Through hands-on gardening, cooking, and nutrition activities, the TX Sprouts intervention was designed to increase children’s exposure and preferences to fruits and vegetables, and as a result would increase intake of those foods and decrease intake of unhealthy energy-dense foods (sugar-sweetened-beverages, chips, cookies, etc.). To our knowledge, our study is the first to examine the impact of participation in a school gardening intervention on dietary quality using the Healthy Eating Index-2015. Compared to the control group, students in the TX Sprouts intervention reported an increase in HEI-2015 total vegetable component scores. We also observed a significant interaction effect between intention-to-treat effects and ethnicity/race for HEI-2015 total vegetables component scores among Non-Hispanic children. Hispanic children in both the intervention and control groups had higher baseline levels of vegetable consumption, possibly explaining why there was no significant increase when stratified by ethnicity/race. Participants in the intervention group also had significantly greater protein intake as a percentage of total energy compared to control. Lastly, although both groups increased their added sugar intake from baseline to follow-up, intake was lower in the intervention group. The intervention had no effect on other aspects of dietary quality or intake; however, changes to these aspects of the diet were not expected because of the intervention. These results reflect the subsample of the larger study sample who completed two 24 h dietary recalls. However, data from the entire sample who completed a dietary screener [42] focusing on intake of fruit and vegetable and sugar-sweetened-beverages found similar results, with increased vegetable consumption in intervention compared to control students [25].

Changes in fruit and vegetable intake are among the most commonly reported outcomes of gardening based interventions [21]. Our results differ from prior school-garden-based RCTs, which found no significant changes in fruit or vegetable consumption in children participating in gardening interventions [14,18,23,24]. Studies of other experimental designs (non-randomized controlled trials and pretest-posttest) have found mixed results, with some finding increases in fruit or vegetable intake [43,44,45,46,47,48] and others finding no change [49,50,51]. Importantly in the aforementioned studies, fruit and vegetable consumption (i.e., amount, frequency, variety) were not operationalized or measured consistently (food frequency questionnaires, visual observation, 24 h dietary recalls), making comparison more challenging. Within our study, while statistically significant, the improvements in vegetable component scores were modest (<1/8 cup), suggesting that the impact of the intervention was limited. However, decreased intake of vegetables was observed in the control group, strengthening the small improvement seen in the intervention group. While the TX Sprouts intervention did not result in significant improvements in dietary intake beyond vegetables or added sugar, it did provide several meaningful insights and important considerations for future garden-based interventions in schools.

Comprehensive, integrated nutrition programs including strategies such as school gardens have been endorsed by several professional organizations as essential components to improve overall health and dietary intake and quality in school children [52,53]. Although TX Sprouts had excellent buy-in from elementary school principals and administrators, many of the schools permitted marketing or the sale of foods and beverages that contradicted the core messages being taught in the TX Sprouts intervention. There is inconclusive evidence that competitive food and beverage sources in schools promote poor dietary intake and some limited evidence to suggest that consumption of these foods is associated with higher Body Mass Index (BMI) and poorer weight outcomes in school children [54,55,56]. School wellness policies can integrate school gardening programs as part of the day-to-day culture to serve as a foundation for promoting health and wellness across a school’s campus. Successful health and wellness policies at the school administrative and district levels can create the mechanism through which school gardens are built and provide the necessary support they need to efficaciously persist [57].

School-based interventions that reach both the child and parent are warranted [58,59]. To strengthen the impact of the TX Sprouts intervention, the study included monthly parent lessons. However, despite numerous promotion strategies and incentives, attendance at these lessons was poor (<7% of participating parents came to one or more parent classes). This created a missed opportunity to connect the lessons that children were learning as part of the intervention with their home environment. Further, research has consistently shown that parent modeling and intake of fruits and vegetables is linked to increased child intake [60]. Children enjoy learning actively and interventions that expose children to fruits and vegetables through hands-on cooking or gardening activities are associated with greater attitudes and preference towards fruits and vegetables and improved dietary intake [16,61,62,63,64,65,66]. Child involvement in home meal preparation specifically is positively associated with improved vegetable preference, self-efficacy for cooking and choosing healthy foods, fruit and vegetable consumption, and overall dietary quality [67,68]. Similarly, involvement with in-home gardening is positively associated with healthy dietary habits, greater fruit and vegetable consumption, and improved mental health and well-being [69]. Exposure to gardening at an early age may even have long-term health benefits. A study of first-year college students found that those who gardened as children had significantly higher reported intakes of fruits and vegetables compared to those who never gardened [70]. Future school-based garden interventions should examine effective methods to increase parental reach as research suggests that an increased level of parental involvement within interventions supports positive dietary behavior change in children [58,59].

While the TX Sprouts study exposed children to fruits and vegetables through either a garden taste-test (seven lessons) or a cooking activity (11 lessons), it did not involve the school food service department as part of the intervention or incorporate garden grown produce as part of school meals. The school gardens in this study occasionally produced excess amounts of produce, allowing us to send vegetables home with students to try with their families, and students were encouraged to try the recipes they learned during their TX Sprouts classes at home. However, our gardens did not produce sufficient amounts of produce to regularly send home produce with students or to make large meals. Future research can examine how partnerships with local farmers and community-supported agriculture (CSA) programs can provide students and their families with locally grown produce. Although research is limited, CSA programs have been linked with reported increased consumption and variety of fruits and vegetables, changes in the household food environment, and changes in meal patterns [71]. A 16-week school-based food co-op program, Brighter Bites, by Sharma et al. (2016) included weekly distribution of fresh produce (~30 pounds; 50–60 servings) as well as nutrition education in schools for children and their parents, and found significant increases in intake of fruits and vegetables [72]. Additionally, a trend that overlaps considerably with school garden programs is the Farm to School (FTS) movement, which seeks to incorporate fresh, locally sourced foods into schools. While FTS activities vary by district, a common strategy is incorporating existing school gardens into programming [73]. Future school-based garden interventions can utilize partnerships and connections with other key stakeholders to create a stronger connection within the school and home environments with the lesson content of school garden programs. This may result in greater improvements in dietary intake among the children.

This study is not able to draw a direct link between availability of vegetables within the home environment and consumption of vegetables and how involvement in the TX Sprouts intervention mediates this relationship. However, research consistently has shown that when healthier foods, such as fruits and vegetables, are available and accessible for purchase by a household and are available and accessible in the home, children have increased intake of vegetables [74,75,76,77]. A study by Wells et al. (2018), found that there were carry-over effects of school gardens on vegetable availability within the home of younger children [78]. Participation in school garden programs such as TX Sprouts may have an impact beyond the school environment and may carry-over into the home environment. Further research is needed, especially in low-income families, to determine how school gardens can support families [77,79] in overcoming barriers to vegetable availability and access within the home environment.

A possible explanation for the results we observed may be a result of changes in psychosocial perceptions. Children may have responded differently to the intervention based on their stage in the Transtheoretical Model of Behavior Change (TTM) [80]. The TTM classifies individuals based on their readiness to change. The model assumes that changes in habitual behaviors occur continuously through a specific process [80]. Additionally, research in adolescents suggests that interventions focusing on intake of specific dietary components show better results [81]. This may explain why we found improvements in vegetable and added sugar consumption, as these were key focus areas of the TX Sprouts intervention but did not see changes to other aspects of the diet with the exception of protein as a percent of total energy.

The main outcome of this study was dietary intake and quality collected from 24 hDRs, which are subject to measurement error, bias, and social desirability [82]. However, when measurement error is taken into consideration during interpretation of data, self-report data remain useful and valuable [83]. Dietary assessment in children poses unique challenges, including potentially limited concept of time, food recognition, knowledge of preparation methods, ability to quantify estimated portion sizes, motivation, literacy, memory capabilities, and concentration span [84,85,86,87]. However, children in our study often completed recalls with the aid of a parent or guardian who could assist with recalling food items and preparation methods or estimating serving sizes. Additionally, dietary assessment data collectors had access to school breakfast and lunch menus for weekday meals, which contained food items and serving sizes. A dietary screener [42] was used to assess fruit and vegetable and sugar sweetened beverage consumption in all children in the study as part of the child survey, and those results are reported elsewhere [25]. Twenty-four-hour dietary recalls are more objective in nature compared to food frequency questionnaires and dietary screeners but are significantly more time-consuming. As a result, 24 hDRs were only conducted on a random subsample of children in intervention and control schools. Lastly, the analytic sample was limited to children who completed recalls at both baseline and post intervention time points and those that had complete baseline demographic data. This sample represents approximately ~15% of consented children included in the clinical trial. These factors in combination may limit the generalizability of the results.

School gardens have been also credited as structural resources that provide gateways to reducing the academic achievement gap in minority and low-income students [88]. However, other research has found that school gardens are more common in schools with a high percentage of white students and a low percentage of students receiving free and reduced-price school lunch [10]. For the TX Sprouts RCT, schools were selected for having a primarily Hispanic and low-socioeconomic background student census [26]. This may limit the generalizability of this study; however, this population was selected by design because Hispanic children are at increased risk for obesity, poor diet quality, and food insecurity [89,90,91].

The dose, intensity, and fidelity of the TX Sprouts intervention may have contributed to the observed positive impact on vegetable intake. All TX Sprouts lessons were taught by well-trained and paid nutrition and gardening educators, in order to control for dosage and reach, and to fully test the effects of the program as designed. However, this does limit the ability to sustain the program moving forward. School gardening programs must address known barriers (e.g., adequate teacher training, existence of a garden leadership committee, and available curriculum) to ensure longevity and success [92].

School gardens were built at schools in the spring between February-May in the spring prior to the academic year of baseline measurements (in the Fall). The garden builds were carried out on a weekend, and the students and their families were invited to participate in building their school’s garden. The build consisted of building physical raised vegetable beds, creating borders around the garden area and laying down soil, mulch, and walkway gravel. During the build, no vegetables, fruits, or other plants were planted. No programming was carried out around the garden prior to the collection of baseline data. Although possible, we do not suspect that this initial activity influenced baseline behaviors or measurements given that it occurred on average 6 months prior.

Due to weather, about one third of garden classes had to be held indoors rather than in the outdoor teaching garden. The full cooking, gardening, and nutrition lesson was still taught indoors on these days, with the exception of activities that required children to actively work with the plants in the garden. However, recent research by our lab group has found that time spent outside of the traditional classroom in a school garden as part of the school day positively enhances direct and indirect academic performance and decreases off-task behaviors (paper in review). The indoor classes may have impacted the effect of the lessons. Our intervention was one academic year (~9 months) in duration, which is longer than most other school garden interventions, often lasting only 10 to 16 weeks [21]; however, this duration may not have been long enough to make significant improvements in areas of dietary intake beyond vegetable intake. These results show that there is a need for interventions that last multiple academic years that build upon lessons taught the prior year as well as longer-term follow-up of school-based garden programs.

## 5. Conclusions

Dietary quality was modestly improved in 3rd–5th grade children following their participation in the TX Sprouts gardening, cooking, and nutrition intervention. Consistent with other studies [21], the intervention was effective at increasing vegetable and decreasing added sugar intake in children. School gardens can play a critical role in shifting children’s perceptions of food and enhancing their access to healthful foods, especially in low-income communities. This study has provided several meaningful insights that can influence the design of future school-based garden studies. Most importantly, there is a need to better engage parents in school-based garden programs as future interventions link the school and home environments, the two places that children spend the majority of their time. One potential strategy to achieve this is through partnerships with local farmers and CSA programs that could provide produce to families to use in meal preparation, allowing the lessons children learned in the school garden to be put into practice with their families in the home environment. Lastly, research is also needed to identify barriers and strategies for sustaining successful school gardening programs, as well as how to maximize their potential reach [92,93,94], in order improve health outcomes in children.

## Figures and Tables

**Table 1:** Repeated Measures ANCOVA of Intent to Treat Effects of a School-Based Cooking, Gardening, and Nutrition Intervention Macronutrients, Shortfall Micronutrients, Micronutrients of Public Health Concern, and Nutrients to Reduce Consumption in Low-Income Elementary Aged Children.

Dietary Components	Control (*n* = 234)			Intervention (*n* = 234)				
	Baseline Mean ± SD	Post Intervention Mean ± SD	Absolute Change Mean ± SD	Baseline Mean ± SD	Post Intervention Mean ± SD	Absolute Change Mean ± SD	Intention to Treat *p*-Value	Intention to Treat x Ethnicity ^1^ Interaction *p*-Value
**Macronutrients**								
Total Energy, kcal	1470 ± 463	1474 ± 486	4.5	1459 ± 641	1476 ± 504	17.6	0.966	0.353
Protein, g/day	58.3 ± 19.1	58.5 ± 21.7	0.1	58.6 ± 26.8	60.6 ± 21.3	1.9	0.305	0.213
Protein % of Energy	16.5 ± 3.9	16.2 ± 3.9	−0.3	16.7 ± 4.0	17.1 ± 4.3	0.4	0.021	0.863
Fat, g/day	57.5 ± 23.2	57.1 ± 23.9	−0.4	56.3 ± 31.2	56.7 ± 22.4	0.4	0.951	0.632
Fat % of Energy	34.0 ± 5.9	33.2 ± 6.3	−0.8	32.9 ± 7.4	33.5 ± 5.6	0.5	0.428	0.991
Carbohydrates, g/day	183.9 ± 63.4	185.8 ± 23.9	1.9	183.9 ± 80.5	185.9 ± 22.4	2.0	0.641	0.690
Carbohydrates % of Energy	49.5 ± 7.6	50.6 ± 7.7	1.1	50.3 ± 8.2	49.4 ± 6.8	−0.9	0.051	0.874
**Shortfall Micronutrients**								
Vitamin A (Retinol Activity Equivalents), mcg/day	433.5 ± 217.5	403.2 ± 188.4	−30.3	389.9 ± 232.5	415.3 ± 198.8	25.5	0.210	0.849
Vitamin E (total alpha-tocopherol), mg/day	5.3 ± 2.7	5.7 ± 2.8	0.4	6.3 ± 8.1	5.8 ± 2.8	−0.5	0.353	0.882
Vitamin C, mg/day	59.3 ± 42.9	62.0 ± 44.8	2.7	68.5 ± 58.1	73.9 ± 63.3	5.4	0.090	0.683
Folate, mcg/day	336.9 ± 161.9	327.1 ± 178.3	−9.8	313.4 ± 181.2	333.2 ± 159.7	19.8	0.384	0.942
Magnesium, mg/day	183.3 ± 63.0	186.6 ± 60.2	3.2	186.1 ± 109.6	195.0 ± 82.0	8.9	0.198	0.721
Iron, mg/day	12.1 ± 5.2	11.7 ± 4.8	−0.4	11.7 ± 5.8	11.8 ± 4.7	0.2	0.563	0.931
**Micronutrients of Public Health Concern**								
Dietary Fiber, g/day	12.5 ± 5.0	12.5 ± 5.6	0.0	12.8 ± 7.5	13.5 ± 7.1	0.7	0.069	0.592
Calcium, mg/day	803.2 ± 329.8	826.9 ± 358.8	23.7	783.7 ± 485.2	841.4 ± 375.3	57.7	0.679	0.746
Vitamin D (calciferol), mcg/day	5.1 ± 3.1	5.1 ± 2.8	−0.1	4.4 ± 2.6	5.1 ± 3.0	0.7	0.359	0.494
Potassium, mg/day	1728 ± 580	1744 ± 584	16.3	1782 ± 807	1859 ± 674.3	77.2	0.089	0.118
**Nutrients to Reduce or Limit Consumption**								
Added Sugar, g/day	38.1 ± 26.4	40.7 ± 25.5	2.6	38.0 ± 23.9	38.4 ± 25.9	0.3	0.050	0.087
Saturated Fat, g/day	20.3 ± 9.3	19.6 ± 9.5	−0.6	19.0 ± 10.8	19.8 ± 8.8	0.7	0.271	0.145
Sodium, mg/day	2513 ± 878	2557 ± 1102	44	2556 ± 1343	2554 ± 955	−1	0.468	0.505

^1^ Ethnicity/race was coded as Hispanic or Non-Hispanic, SD = Standard Deviation.

**Table 2:** Repeated Measures ANCOVA of Intent to Treat Effects of a School-Based Cooking, Gardening, and Nutrition Intervention on Healthy Eating Index—2015 Total and Component Scores in Low-Income Elementary Aged Children.

Variable	Control (*n* = 234)			Intervention (*n* = 234)				
	Baseline Mean ± SD	Post Intervention Mean ± SD	Absolute Change Mean ± SD	Baseline Mean ± SD	Post Intervention Mean ± SD	Absolute Change Mean ± SD	Intention to Treat *p*-Value	Intention to Treat x Ethnicity ^1^ Interaction *p*-Value
HEI Total Score	53 (13.1)	54 (12.9)	1 (16.1)	52.8 (11.8)	54.9 (13.4)	2 (14.8)	0.380	0.633
Total Vegetables	2.5 (1.4)	2.4 (1.5)	−0.1 (1.8)	2.7 (1.6)	2.9 (1.5)	0.2 (2.0)	0.003	0.033
Greens and Beans	1.9 (2.1)	1.6 (2.1)	−0.3 (2.7)	1.8 (2.1)	1.9 (2.2)	0.1 (2.7)	0.061	0.421
Total Fruit	2.4 (1.7)	2.6 (1.8)	0.2 (2.3)	2.7 (1.9)	2.8 (1.9)	0.1 (2.1)	0.490	0.184
Whole Fruit	2.5 (1.9)	2.6 (2.0)	0.1 (2.5)	2.7 (2.1)	2.7 (2.1)	−0.1 (2.5)	0.932	0.924
Whole Grains	4.8 (3.6)	5.2 (3.5)	0.3 (4.9)	4.1 (3.4)	4.6 (3.6)	0.4 (4.6)	0.090	0.432
Total Dairy	7.5 (2.7)	7.5 (2.8)	−0.1 (3.5)	6.9 (3.2)	7.6 (2.7)	0.6 (3.5)	0.498	0.500
Total Protein	4.3 (1.1)	4.4 (1.1)	0.1 (1.6)	4.5 (1.0)	4.5 (1.0)	0.0 (1.3)	0.269	0.304
Seafood and Plant Proteins	2 (2.2)	2.1 (2.3)	0.1 (2.9)	2.2 (2.3)	2.4 (2.3)	0.3 (2.8)	0.125	0.312
Fatty Acids	3.7 (3.2)	4.2 (3.3)	0.5 (4.3)	4.1 (3.4)	3.8 (3.1)	−0.3 (4.2)	0.171	0.681
Sodium ^2^	3.6 (3.1)	3.8 (3.1)	0.2 (4.1)	3.4 (3.0)	3.5 (3.0)	0.1 (3.9)	0.308	0.396
Refined Grains ^2^	4.6 (3.6)	4.7 (3.5)	0.1 (4.7)	4.2 (3.5)	5.1 (3.6)	0.9 (4.5)	0.100	0.641
Added Sugar ^2^	7.9 (2.4)	7.3 (2.8)	−0.5 (3.5)	7.7 (2.3)	7.8 (2.3)	0.1 (2.8)	0.068	0.087
Saturated Fat ^2^	5.1 (3.1)	5.6 (3.3)	0.5 (4.2)	5.8 (3.2)	5.4 (3.0)	−0.4 (4.2)	0.400	0.563

^1^ Ethnicity/race was coded as Hispanic or Non-Hispanic, ^2^ Healthy Eating Index-2015 Moderation Components are reversed scored—a higher score represents lower intake, SD = Standard Deviation.

## Data Availability

The data that support the findings of this study are available from the corresponding author (J.N.D.), upon reasonable request.

## References

[1] U.S. Department of Agriculture, U.S. Department of Health and Human Services (2020). Dietary Guidelines for Americans, 2020–2025.

[2] Scaglioni S., De Cosmi V., Ciappolino V., Parazzini F., Brambilla P., Agostoni C. (2018). Factors influencing children’s eating behaviours. Nutrients.

[3] Liu J., Rehm C.D., Onopa J., Mozaffarian D. (2020). Trends in diet quality among youth in the United States, 1999–2016. JAMA.

[4] Dunford E., Popkin B. (2018). 37 year snacking trends for US children 1977–2014. Pediatr. Obes..

[5] Bleich S.N., Vercammen K.A., Koma J.W., Li Z. (2018). Trends in beverage consumption among children and adults, 2003–2014. Obesity.

[6] Rehm C.D., Drewnowski A. (2016). Trends in consumption of solid fats, added sugars, sodium, sugar-sweetened beverages, and fruit from fast food restaurants and by fast food restaurant type among US children, 2003–2010. Nutrients.

[7] Thomson J.L., Tussing-Humphreys L.M., Goodman M.H., Landry A.S. (2019). Diet quality in a nationally representative sample of American children by sociodemographic characteristics. Am. J. Clin. Nutr..

[8] Cullen K.W., Chen T.-A. (2017). The contribution of the USDA school breakfast and lunch program meals to student daily dietary intake. Prev. Med. Rep..

[9] Division of Population Health National Center for Chronic Disease Prevention and Health Promotion National Health Education Standards. https://www.cdc.gov/healthyschools/sher/standards/index.htm.

[10] Turner L., Eliason M., Sandoval A., Chaloupka F.J. (2016). Increasing prevalence of US elementary school gardens, but disparities reduce opportunities for disadvantaged students. J. Sch. Health.

[11] Schneider S., Pharr J., Bungum T. (2017). Impact of school garden participation on the health behaviors of children. Health Behav. Policy Rev..

[12] Lam V., Romses K., Renwick K. (2019). Exploring the relationship between school gardens, food literacy and mental well-being in youth using photovoice. Nutrients.

[13] Davis J.N., Martinez L.C., Spruijt-Metz D., Gatto N.M. (2016). LA Sprouts: A 12-week gardening, nutrition, and cooking randomized control trial improves determinants of dietary behaviors. J. Nutr. Educ. Behav..

[14] Gatto N., Martinez L., Spruijt-Metz D., Davis J. (2017). LA Sprouts randomized controlled nutrition, cooking and gardening programme reduces obesity and metabolic risk in Hispanic/Latino youth. Pediatr. Obes..

[15] Berezowitz C.K., Bontrager Yoder A.B., Schoeller D.A. (2015). School gardens enhance academic performance and dietary outcomes in children. J. Sch. Health.

[16] Landry M.J., Markowitz A.K., Asigbee F.M., Gatto N.M., Spruijt-Metz D., Davis J.N. (2019). Cooking and gardening behaviors and improvements in dietary intake in hispanic/latino youth. Child. Obes..

[17] Wells N.M., Myers B.M., Henderson C.R. (2014). School gardens and physical activity: A randomized controlled trial of low-income elementary schools. Prev. Med..

[18] Van den Berg A., Warren J.L., McIntosh A., Hoelscher D., Ory M.G., Jovanovic C., Lopez M., Whittlesey L., Kirk A., Walton C. (2020). Impact of a gardening and physical activity intervention in Title 1 schools: The TGEG study. Child. Obes..

[19] Robinson-O’Brien R., Story M., Heim S. (2009). Impact of garden-based youth nutrition intervention programs: A review. J. Am. Diet. Assoc..

[20] Ozer E.J. (2007). The effects of school gardens on students and schools: Conceptualization and considerations for maximizing healthy development. Health Educ. Behav..

[21] Savoie-Roskos M.R., Wengreen H., Durward C. (2017). Increasing fruit and vegetable intake among children and youth through gardening-based interventions: A systematic review. J. Acad. Nutr. Diet..

[22] Skelton K.R., Lowe C., Zaltz D.A., Benjamin-Neelon S.E. (2020). Garden-based interventions and early childhood health: An umbrella review. Int. J. Behav. Nutr. Phys. Act..

[23] Christian M.S., Evans C.E., Nykjaer C., Hancock N., Cade J.E. (2014). Evaluation of the impact of a school gardening intervention on children’s fruit and vegetable intake: A randomised controlled trial. Int. J. Behav. Nutr. Phys. Act..

[24] Brouwer R.J.N., Neelon S.E.B. (2013). Watch Me Grow: A garden-based pilot intervention to increase vegetable and fruit intake in preschoolers. BMC Public Health.

[25] Davis J.N., Pérez A., Asigbee F.M., Landry M.J., Vandyousefi S., Ghaddar R., Hoover A., Jeans M., Nikah K., Fischer B. (2021). School-based gardening, cooking and nutrition intervention increased vegetable intake but did not reduce BMI: Texas sprouts-a cluster randomized controlled trial. Int. J. Behav. Nutr. Phys. Act..

[26] Davis J.N., Nikah K., Asigbee F.M., Landry M.J., Vandyousefi S., Ghaddar R., Hoover A., Jeans M., Pont S.J., Richards D. (2019). Design and participant characteristics of TX sprouts: A school-based cluster.randomized gardening, nutrition, and cooking intervention. Contemp. Clin. Trials.

[27] McLeroy K.R., Bibeau D., Steckler A., Glanz K. (1988). An ecological perspective on health promotion programs. Health Educ. Q.

[28] Martinez L.C., Gatto N.M., Spruijt-Metz D., Davis J.N. (2015). Design and methodology of the LA Sprouts nutrition, cooking and gardening program for Latino youth: A randomized controlled intervention. Contemp. Clin. Trials.

[29] Texas A&M Agrilife Extension Service (2015). Learn, Grow, Eat, and Go!.

[30] Centers for Disease Control and Prevention (CDC) (2011). School health guidelines to promote healthy eating and physical activity. MMWR. Recomm. Rep. Morb. Mortal. Wkly. Report. Recomm. Rep..

[31] Feskanich D., Sielaff B.H., Chong K., Buzzard I.M. (1989). Computerized collection and analysis of dietary intake information. Comput. Methods Programs Biomed..

[32] Johnson R.K., Driscoll P., Goran M.I. (1996). Comparison of multiple-pass 24-hour recall estimates of energy intake with total energy expenditure determined by the doubly labeled water method in young children. J. Am. Diet. Assoc..

[33] U.S. Department of Health and Human Services, U.S. Department of Agriculture (2015). Dietary Guidelines for Americans 2015–2020.

[34] Reedy J., Lerman J.L., Krebs-Smith S.M., Kirkpatrick S.I., Pannucci T.E., Wilson M.M., Subar A.F., Kahle L.L., Tooze J.A. (2018). Evaluation of the healthy eating index—2015. J. Acad. Nutr. Diet..

[35] Kirkpatrick S.I., Reedy J., Krebs-Smith S.M., Pannucci T.E., Subar A.F., Wilson M.M., Lerman J.L., Tooze J.A. (2018). Applications of the healthy eating index for surveillance, epidemiology, and intervention research: Considerations and caveats. J. Acad. Nutr. Diet..

[36] Krebs-Smith S.M., Pannucci T.E., Subar A.F., Kirkpatrick S.I., Lerman J.L., Tooze J.A., Wilson M.M., Reedy J. (2018). Update of the healthy eating index: HEI-2015. J. Acad. Nutr. Diet..

[37] Nutrition Coordinating Center Healthy Eating Index. http://www.ncc.umn.edu/healthy-eating-index-hei/.

[38] Dietary Guidelines Advisory Committee (2020). Scientific Report of the 2020 Dietary Guidelines Advisory Committee: Advisory Report to the Secretary of Agriculture and the Secretary of Health and Human Services.

[39] Harris P.A., Taylor R., Minor B.L., Elliott V., Fernandez M., O’Neal L., McLeod L., Delacqua G., Delacqua F., Kirby J. (2019). The REDCap consortium: Building an international community of software platform partners. J. Biomed. Inform..

[40] Harris P.A., Taylor R., Thielke R., Payne J., Gonzalez N., Conde J.G. (2009). Research electronic data capture (REDCap)—A metadata-driven methodology and workflow process for providing translational research informatics support. J. Biomed. Inform..

[41] Murray D.M., Blitstein J.L. (2003). Methods to reduce the impact of intraclass correlation in group-randomized trials. Eval. Rev..

[42] Landry M., Ranjit N., Hoelscher D., Asigbee F., Vandyousefi S., Ghaddar R., Davis J. (2019). Validity and reliability of an expanded vegetable questionnaire among elementary school children. Curr. Dev. Nutr..

[43] Castro D.C., Samuels M., Harman A.E. (2013). Growing healthy kids: A community garden–based obesity prevention program. Am. J. Prev. Med..

[44] McAleese J.D., Rankin L.L. (2007). Garden-based nutrition education affects fruit and vegetable consumption in sixth-grade adolescents. J. Am. Diet. Assoc..

[45] Duncan M.J., Eyre E., Bryant E., Clarke N., Birch S., Staples V., Sheffield D. (2015). The impact of a school-based gardening intervention on intentions and behaviour related to fruit and vegetable consumption in children. J. Health Psychol..

[46] Ratcliffe M.M., Merrigan K.A., Rogers B.L., Goldberg J.P. (2011). The effects of school garden experiences on middle school-aged students’ knowledge, attitudes, and behaviors associated with vegetable consumption. Health Promot. Pract..

[47] Parmer S.M., Salisbury-Glennon J., Shannon D., Struempler B. (2009). School gardens: An experiential learning approach for a nutrition education program to increase fruit and vegetable knowledge, preference, and consumption among second-grade students. J. Nutr. Educ. Behav..

[48] Lautenschlager L., Smith C. (2007). Understanding gardening and dietary habits among youth garden program participants using the Theory of Planned Behavior. Appetite.

[49] Davis J.N., Ventura E.E., Cook L.T., Gyllenhammer L.E., Gatto N.M. (2011). LA Sprouts: A gardening, nutrition, and cooking intervention for Latino youth improves diet and reduces obesity. J. Am. Diet. Assoc..

[50] Morgan P.J., Warren J.M., Lubans D.R., Saunders K.L., Quick G.I., Collins C.E. (2010). The impact of nutrition education with and without a school garden on knowledge, vegetable intake and preferences and quality of school life among primary-school students. Public Health Nutr..

[51] Meinen A., Friese B., Wright W., Carrel A. (2012). Youth gardens increase healthy behaviors in young children. J. Hunger Environ. Nutr..

[52] Hayes D., Contento I.R., Weekly C. (2018). Position of the academy of nutrition and dietetics, society for nutrition education and behavior, and school nutrition association: Comprehensive nutrition programs and services in schools. J. Acad. Nutr. Diet..

[53] Mozaffarian D., Afshin A., Benowitz N.L., Bittner V., Daniels S.R., Franch H.A., Jacobs D.R., Kraus W.E., Kris-Etherton P.M., Krummel D.A. (2012). Population approaches to improve diet, physical activity, and smoking habits: A scientific statement from the American Heart Association. Circulation.

[54] Chriqui J.F., Pickel M., Story M. (2014). Influence of school competitive food and beverage policies on obesity, consumption, and availability: A systematic review. JAMA Pediatr..

[55] Velazquez C.E., Black J.L., Potvin Kent M. (2017). Food and beverage marketing in schools: A review of the evidence. Int. J. Environ. Res. Public Health.

[56] Sildén K.E. (2018). Impact of competitive foods in public schools on child nutrition: Effects on adolescent obesity in the United States an integrative systematic literature review. Glob. Health Action.

[57] Burt K.G., Koch P., Contento I. (2017). Development of the GREEN (Garden Resources, Education, and Environment Nexus) tool: An evidence-based model for school garden integration. J. Acad. Nutr. Diet..

[58] Van Lippevelde W., Verloigne M., De Bourdeaudhuij I., Brug J., Bjelland M., Lien N., Maes L. (2012). Does parental involvement make a difference in school-based nutrition and physical activity interventions? A systematic review of randomized controlled trials. Int. J. Public Health.

[59] Muzaffar H., DiFilippo K.N., Fitzgerald N., Tidwell D.K., Idris R., Kurzynske J.S., Chapman-Novakofski K. (2020). A systematic review of interventions to improve diet quality of children that included parents versus those without parental involvement. Curr. Dev. Nutr..

[60] Pearson N., Biddle S.J., Gorely T. (2009). Family correlates of fruit and vegetable consumption in children and adolescents: A systematic review. Public Health Nutr..

[61] Asigbee F.M., Davis J.N., Markowitz A.K., Landry M.J., Vandyousefi S., Ghaddar R., Ranjit N., Warren J., van den Berg A. (2020). The association between child cooking involvement in food preparation and fruit and vegetable intake in a hispanic youth population. Curr. Dev. Nutr..

[62] Allirot X., da Quinta N., Chokupermal K., Urdaneta E. (2016). Involving children in cooking activities: A potential strategy for directing food choices toward novel foods containing vegetables. Appetite.

[63] Overcash F., Ritter A., Mann T., Mykerezi E., Redden J., Rendahl A., Vickers Z., Reicks M. (2018). Impacts of a vegetable cooking skills program among low-income parents and children. J. Nutr. Educ. Behav..

[64] Van der Horst K., Ferrage A., Rytz A. (2014). Involving children in meal preparation. Effects on food intake. Appetite.

[65] Evans A., Ranjit N., Fair C.N., Jennings R., Warren J.L. (2016). Previous gardening experience and gardening enjoyment is related to vegetable preferences and consumption among low-income elementary school children. J. Nutr. Educ. Behav..

[66] Prescott M.P., Lohse B., Mitchell D.C., Cunningham-Sabo L. (2019). Child assessments of vegetable preferences and cooking self-efficacy show predictive validity with targeted diet quality measures. BMC Nutr..

[67] Quelly S.B. (2019). Helping with meal preparation and children’s dietary intake: A literature review. J. Sch. Nurs..

[68] Ng C.M., Satvinder K., Koo H.C., Yap R.W.K., Mukhtar F. (2020). Influences of psychosocial factors and home food availability on healthy meal preparation. Matern. Child. Nutr..

[69] Van Lier L.E., Utter J., Denny S., Lucassen M., Dyson B., Clark T. (2017). Home gardening and the health and well-being of adolescents. Health Promot. Pract..

[70] Loso J., Staub D., Colby S.E., Olfert M.D., Kattelmann K., Vilaro M., Colee J., Zhou W., Franzen-Castle L., Mathews A.E. (2018). Gardening experience is associated with increased fruit and vegetable intake among first-year college students: A cross-sectional examination. J. Acad. Nutr. Diet..

[71] Vasquez A., Sherwood N.E., Larson N., Story M. (2017). Community-supported agriculture as a dietary and health improvement strategy: A narrative review. J. Acad. Nutr. Diet..

[72] Sharma S.V., Markham C., Chow J., Ranjit N., Pomeroy M., Raber M. (2016). Evaluating a school-based fruit and vegetable co-op in low-income children: A quasi-experimental study. Prev. Med..

[73] Prescott M.P., Cleary R., Bonanno A., Costanigro M., Jablonski B.B., Long A.B. (2020). Farm to school activities and student outcomes: A systematic review. Adv. Nutr..

[74] Poulsen M.N., Bailey-Davis L., Pollak J., Hirsch A.G., Schwartz B.S. (2019). Household food insecurity and home food availability in relation to youth diet, body mass index, and adiposity. J. Acad. Nutr. Diet..

[75] Jago R., Baranowski T., Baranowski J.C. (2007). Fruit and vegetable availability: A micro environmental mediating variable?. Public Health Nutr..

[76] Cook L.T., O’Reilly G.A., DeRosa C.J., Rohrbach L.A., Spruijt-Metz D. (2015). Association between home availability and vegetable consumption in youth: A review. Public Health Nutr..

[77] Mook K., Laraia B.A., Oddo V.M., Jones-Smith J.C. (2016). Food security status and barriers to fruit and vegetable consumption in two economically deprived communities of Oakland, California, 2013–2014. Prev. Chronic Dis..

[78] Wells N.M., Meyers B.M., Todd L.E., Henderson C.R., Barale K., Gaolach B., Ferenz G., Aitken M., Caroline C.T., Pattison K.O. (2018). The carry-over effects of school gardens on fruit and vegetable availability at home: A randomized controlled trial with low-income elementary schools. Prev. Med..

[79] Landry M.J., Burgermaster M., van den Berg A.E., Asigbee F.M., Vandyousefi S., Ghaddar R., Jeans M.R., Yau A., Davis J.N. (2020). Barriers to preparing and cooking vegetables are associated with decreased home availability of vegetables in low-income households. Nutrients.

[80] Prochaska J.O., Johnson S., Lee P. (2009). The transtheoretical model of behavior change. Am. J. Health Promot..

[81] Nakabayashi J., Melo G.R.-I., Toral N. (2020). Transtheoretical model-based nutritional interventions in adolescents: A systematic review. BMC Public Health.

[82] Thompson F.E., Subar A.F. (2017). Dietary assessment methodology. Nutrition in the Prevention and Treatment of Disease.

[83] Subar A.F., Freedman L.S., Tooze J.A., Kirkpatrick S.I., Boushey C., Neuhouser M.L., Thompson F.E., Potischman N., Guenther P.M., Tarasuk V. (2015). Addressing current criticism regarding the value of self-report dietary data. J. Nutr..

[84] McPherson R.S., Hoelscher D.M., Alexander M., Scanlon K.S., Serdula M.K. (2000). Dietary assessment methods among school-aged children: Validity and reliability. Prev. Med..

[85] Foster E., Bradley J. (2018). Methodological considerations and future insights for twenty-four hour dietary recall assessment in children. Nutr. Res..

[86] Livingstone M., Robson P. (2000). Measurement of dietary intake in children. Proc. Nutr. Soc..

[87] Livingstone M., Robson P., Wallace J. (2004). Issues in dietary intake assessment of children and adolescents. Br. J. Nutr..

[88] Ray R., Fisher D.R., Fisher-Maltese C. (2016). School gardens in the city: Does environmental equity help close the achievement gap?. Du Bois Rev..

[89] Ogden C.L., Fryar C.D., Martin C.B., Freedman D.S., Carroll M.D., Gu Q., Hales C.M. (2020). Trends in obesity prevalence by race and hispanic origin—1999–2000 to 2017–2018. JAMA.

[90] Coleman-Jensen A., Rabbitt M.P., Gregory C.A., Singh A. (2020). Household Food Security in the United States in 2019.

[91] Landry M.J., van den Berg A.E., Asigbee F.M., Vandyousefi S., Ghaddar R., Davis J.N. (2019). Child-report of food insecurity is associated with diet quality in children. Nutrients.

[92] Hoover A., Vandyousefi S., Martin B., Nikah K., Cooper M.H., Muller A., Marty E., Duswalt-Epstein M., Burgermaster M., Waugh L. (2021). Barriers, Strategies, and Resources to Thriving School Gardens. J. Nutr. Educ. Behav..

[93] Burt K.G., Luesse H.B., Rakoff J., Ventura A., Burgermaster M. (2018). School gardens in the United States: Current barriers to integration and sustainability. Am. J. Public Health.

[94] Davis J.N., Spaniol M.R., Somerset S. (2015). Sustenance and sustainability: Maximizing the impact of school gardens on health outcomes. Public Health Nutr..

